# Causal relationship between ankylosing spondylitis and multiple sclerosis: Evidence from 2-sample Mendelian randomization

**DOI:** 10.1097/MD.0000000000044788

**Published:** 2025-09-19

**Authors:** Rui Liu, Gang Peng, Yongbing Xiao

**Affiliations:** a Department of Orthopedics, Xiangya Hospital of Central South University, Changsha, Hunan Province, China; b School of Mathematics and Statistics, Central South University, Changsha, Hunan Province, China.

**Keywords:** ankylosing spondylitis, Mendelian randomization, multiple sclerosis

## Abstract

Ankylosing spondylitis (AS) and multiple sclerosis (MS) are chronic immune-mediated disorders with overlapping epidemiological and genetic features, but their causal relationship remains unclear. In this study, we performed a 2-sample Mendelian randomization (MR) analysis using large-scale genome-wide association study summary statistics from European populations to investigate whether AS causally influences the risk of MS. Instrumental variables were selected based on stringent significance, independence, and validity criteria. The primary MR analyses, including the inverse variance weighted method, indicated that AS is associated with an increased risk of MS (odds ratio = 2.18, 95% confidence interval: 1.31–3.63, *P* = .00), and this finding was supported by the MR Egger method. Sensitivity analyses confirmed the robustness of the results. Inverse MR analysis found no evidence for a causal effect of MS on AS. These findings contribute to the understanding of the shared genetic architecture and pathophysiological pathways between AS and MS. Further studies in more diverse populations and with nonlinear models are warranted to validate and extend these findings.

## 1. Introduction

Multiple sclerosis (MS) is a chronic inflammatory and neurodegenerative condition that manifests mostly in young adults between the ages of 20 and 30 years.^[1]^ Globally, MS affects an estimated 2.8 million individuals.^[2]^ It is caused by focal inflammatory lesions and wide-spread low-grade inflammation in the brain and spinal cord.^[3,4]^ The clinical presentation of MS is diverse, encompassing neurological symptoms such as visual disturbances, motor weakness, cognitive impairments, and sensory deficits.^[5]^ The exact etiology of MS remains unresolved, though it is widely believed to arise from a combination of genetic predisposition and environmental factors that provoke an autoimmune response against myelin.^[6]^ Several genes, particularly those involved in immune regulation, have been identified as contributing to MS susceptibility. Additionally, environmental factors, including viral infections and vitamin D deficiency, have been implicated in the onset and progression of MS.^[7]^ Despite significant advancements in understanding its pathophysiology, current therapeutic strategies primarily focus on managing relapses and slowing disease progression, with no curative treatments available.^[8]^ Thus, identifying modifiable risk factors for MS is crucial to improve preventive and therapeutic approaches.

Ankylosing spondylitis (AS) is a chronic inflammatory disease predominantly affecting the spine and sacroiliac joints, leading to progressive stiffness and pain. As a subtype of spondyloarthritis,^[9,10]^ AS typically manifests in young adults, with onset generally occurring between the ages of 15 and 30 years.^[11]^ According to a systematic review of 36 studies, AS affects an estimated 1.30 to 1.56 million individuals in Europe and 4.63 to 4.98 million in Asia.^[12]^ The disease is characterized by persistent inflammation of the axial skeleton, particularly the sacroiliac joints, which can eventually lead to spinal fusion and significant functional impairment. In addition to its axial involvement, AS is frequently associated with extra-articular manifestations, including uveitis, psoriasis, and inflammatory bowel disease, contributing to its systemic nature.^[13]^ The pathogenesis of AS is complex, involving genetic predisposition, environmental triggers, and immune system dysregulation, with the HLA-B27 gene identified as a major genetic risk factor for the disease.^[14,15]^ Despite advances in understanding its underlying mechanisms, current treatment options remain limited, primarily addressing symptom management with nonsteroidal anti-inflammatory drugs and biologics.^[16–18]^ However, these therapies do not prevent disease progression, underscoring the urgent need for research into modifiable factors that may influence disease course.

The relationship between AS and MS has been explored in various studies, with some suggesting a potential association between these 2 inflammatory conditions. Both diseases share common features, including immune system dysregulation, and may involve overlapping genetic and environmental risk factors.^[15]^ Some studies have found a higher prevalence of MS in patients with AS, implying a shared pathophysiological pathway.^[19,20]^ As it is known, AS is closely associated with the *HLA-B27* gene.^[6]^ Meanwhile, the *HLA* gene cluster in the polymorphic MHC locus is considered a major genetic MS risk factor, with notable variants in class II *HLA-DRB1* (e.g., HLA-DRB1*15:01) and *HLA-DPB1* genes and class I *HLA-A* and *HLA-B* genes, suggesting a potential link between AS and MS.^[21]^ In 1989, A Calin^[22]^ hypothesized the possible comorbidity of MS and AS, raising the question of whether there is a genetic association between MS and AS. In recent years, an increasing body of research has supported this hypothesis and revealed potential immunological connections between the 2 diseases. Orhan Kucuksahin et al^[23]^ used Secukinumab, an IL-17 inhibitor, to treat patients with both MS and AS and observed the alleviation of symptoms in both diseases, suggesting that the IL-17 pathway might play a role in the shared pathological processes of AS and MS. This finding provides new evidence for the immunological association between the 2 diseases. However, despite clinical co-occurrence in certain individuals, the causal relationship between AS and MS remains poorly understood.^[22]^ Moreover, traditional epidemiological methods like cohort and case–control studies offer insights into the AS–MS relationship but are limited by confounding, inverse causality, and selection bias, making it difficult to establish the causal direction between these conditions.^[24]^ Mendelian randomization (MR) addresses these issues by using genetic variants as instrumental variables (IVs) to infer causality, leveraging their random allocation at conception to minimize confounding and inverse causality.^[20]^ Two-sample MR further strengthens this approach by using separate datasets for AS and MS, enhancing statistical power and reducing bias, thus offering a more reliable assessment of whether AS causally influences MS.

## 2. Methods

### 2.1. Data sources

Table 1 outlines the detailed genome-wide association study (GWAS) information used in the present study. The GWAS data for AS were sourced from the study by Matthew A. Brown et al, which included 10,619 cases and 15,145 controls, with the majority of the participants of European descent.^[25]^ The GWAS data for MS were also of European ancestry, including 4888 cases and 10,395 controls.^[26]^ Since this study is based on existing publications and public databases, ethical approval is not required.

**Table 1:** Details of genome-wide association study data used in the analysis.

Phenotype	Sample size	Population	Source	Journal	References (PMID)
Multiple sclerosis	15,283	European	German Competence Network Multiple Sclerosis project	Science Advances	27386562
Ankylosing spondylitis	25,764	European	Wellcome Trust Case Control Consortium 2 project	Nature Genetics	23749187

PMID = PubMed identifier.

### 2.2. Study design

**Figure 1.**
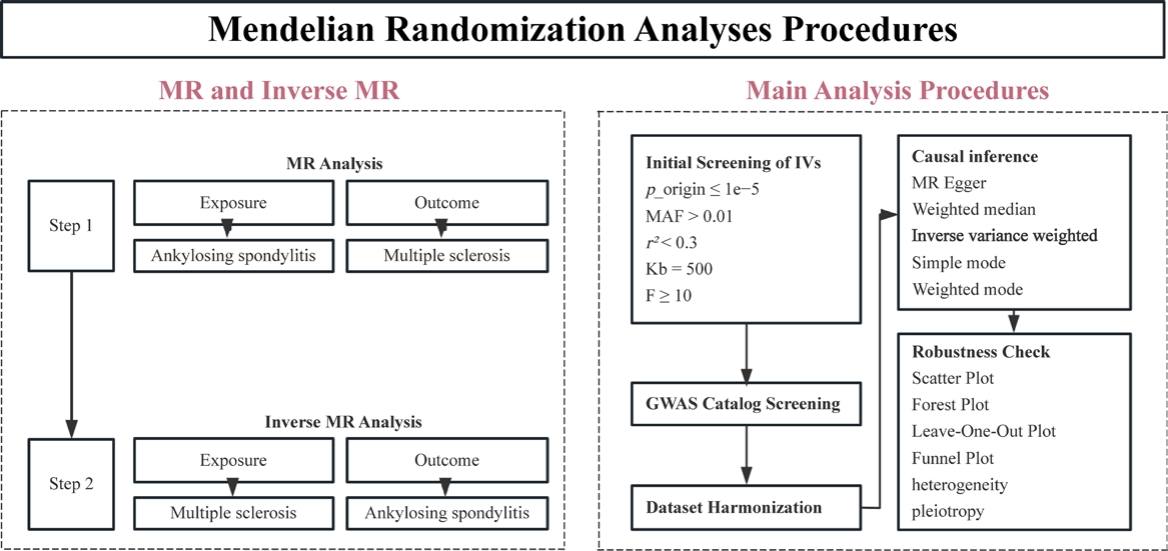
Mendelian randomization (MR) analysis procedures. This figure illustrates the MR analysis workflow, including both forward and inverse MR approaches. The analysis involves the initial screening of instrumental variables based on *P*-value < 1 × 10⁻⁵, minor allele frequency (MAF) > 0.01, an *r²* threshold < 0.3, and a 500 kb window to reduce linkage disequilibrium (LD). SNPs with an *F*-statistic > 10 were retained for adequate statistical power. IVs were further screened by manually searching the GWAS catalog database and removing SNPs associated with confounders. Causal inference was performed using 5 MR methods. The robustness of the results was assessed using scatter, forest, leave-one-out, and funnel plots, as well as tests for heterogeneity and pleiotropy. IVs = instrumental variables, IVW = inverse variance weighted, MR = Mendelian randomization.

Figure 1 depicts the overall study design. Initially, we investigated the causal relationship between AS (exposure) and MS (outcome). Subsequently, we explored the potential existence of inverse causality. All statistical analyses were performed using R version 4.4.0 (The R Foundation for Statistical Computing, Vienna, Austria), utilizing the TwoSampleMR,^[27]^ MR-PRESSO,^[28]^ and forestplot packages.

### 2.3. IVs selection

We strictly adhered to the 3 core assumptions for selecting IVs when choosing single nucleotide polymorphisms (SNPs): relevance; independence; exclusivity. These assumptions ensure that the causal path from SNPs to the outcome variable is mediated solely through the exposure. In the GWAS, SNPs associated with exposure were selected based on a significance threshold for genome-wide analysis (*P* < 1 × 10⁻⁵) and a minor allele frequency >0.01. To reduce linkage disequilibrium, we referenced the European Thousand Genomes Project, applying an *r*^*2*^ threshold of <0.3 and using a 500 kb window for linkage disequilibrium assessment. Only SNPs with an *F*-statistic >10 were retained to ensure sufficient statistical power. To obviate confounding SNPs, we searched and filtered SNPs related to other diseases or phenotypes in the GWASCATALOG website (https://www.ebi.ac.uk/gwas/) to further reduce potential confounding effects. This process ensured that the selected SNPs were only associated with the exposure (AS) and not influenced by other diseases (such as MS-related risk factors).

### 2.4. Main analysis

We harmonized the exposure data (AS) and outcome data (MS) to ensure consistency in SNPs, effect alleles, standard errors, and *P*-values. To ensure the alignment of effect directions between the exposure and outcome variables, we used the harmonise_data function, which helped eliminate potential biases arising from data inconsistencies. Then, we employed 5 methods as the primary analytical tools (MR Egger, weighted median, inverse variance weighted, simple mode, and weighted mode) to evaluate the causal relationship between AS and MS. These methods aggregate effect estimates from multiple IVs, providing robust causal estimates.

### 2.5. Robustness test

We performed 4 diagnostic plots to assess the robustness of our MR findings: the scatter plot visualizes the relationship between genetic instruments and both the exposure and outcome, providing an initial check for linearity and variation. Forest plot displays causal estimates from individual IVs along with their confidence intervals (CIs), allowing for a comparison of effect sizes and heterogeneity across IVs. The leave-one-out plot examines the influence of each SNP on the overall causal estimate by removing 1 IV at a time, highlighting any influential SNPs. The funnel plot checks for publication bias or unmeasured confounding by displaying the effect sizes against their standard errors. Symmetry suggests no bias, while asymmetry may indicate potential issues.

We assessed heterogeneity using Cochran *Q* statistic, considering a *P*-value below .05 as indicative of significant variability. Identifying significant heterogeneity is crucial, as it may suggest differences in effect sizes across various IVs, which could impact the accuracy of the overall causal inference. To detect and correct for pleiotropy, we employed Egger regression. This method tests for pleiotropy by examining whether the regression intercept significantly deviates from zero, with a non-zero intercept indicating the presence of pleiotropy.

## 3. Results

### 3.1. MR and inverse MR

**Figure 2.**
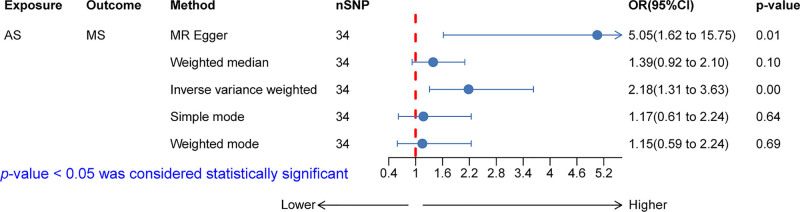
Gene-level causal relationship between ankylosing spondylitis (AS) and multiple sclerosis (MS). The IVW method yielded an odds ratio (OR) of 2.18 (95% CI: 1.31–3.63) with a *P*-value <.05, indicating a significant causal relationship between AS and MS at the genetic level. This suggests that AS may be a genetic risk factor for MS. The IVW method is considered the most reliable as it combines estimates from multiple instrumental variables, weighting each based on precision to provide an optimal and unbiased effect estimate. Additionally, the MR Egger method also showed a significant positive correlation between AS and MS, with a *P*-value <.05. AS = ankylosing spondylitis, IVW = inverse variance weighted, MS = multiple sclerosis, SNP = single nucleotide polymorphism.

As shown in Figure 2, a total of 34 SNPs were used in the analysis, and the inverse variance weighted (IVW) method yielded an odds ratio (OR) of 2.18 (95% CI: 1.31–3.63) with a *P*-value <.05, indicating a significant causal relationship between the exposure and the outcome at the genetic level, suggesting that AS may be a risk factor for MS. The IVW method is considered the most reliable because it combines multiple IV estimates by weighting each based on precision, providing the most optimal and unbiased effect estimate. Additionally, the MR Egger method also supported this result, showing a significant positive correlation between the exposure and the outcome, with *P*-values <.05. However, the weighted median, simple mode, and weighted mode methods did not detect a significant relationship.

**Figure 3.**
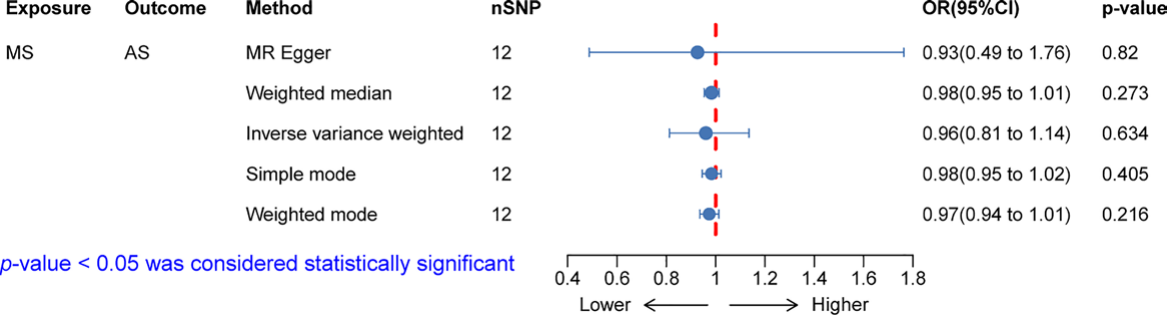
Inverse Mendelian randomization (MR) analysis. The results of the inverse MR analysis, which included MR Egger (OR: 0.93, *P* = .82), weighted median (OR: 0.98, *P* = .273), inverse variance weighted (IVW) (OR: 0.96, *P* = .634), simple mode (OR: 0.98, *P* = .405), and weighted mode (OR: 0.97, *P* = .216). All methods yielded *P*-values >.05, indicating no evidence of a causal relationship between MS and AS at the genetic level. AS = ankylosing spondylitis, MS = multiple sclerosis, OR = odds ratio.

As illustrated in Figure 3, the inverse MR analysis, including MR Egger (OR: 0.93, *P* = .82), weighted median (OR: 0.98, *P* = .273), IVW (OR: 0.96, *P* = .634), simple mode (OR: 0.98, *P* = .405), and weighted mode (OR: 0.97, *P* = .216), showed *P*-values >.05, indicating no evidence of a causal relationship between MS and AS at the genetic level.

### 3.2. Robustness test

**Figure 4.**
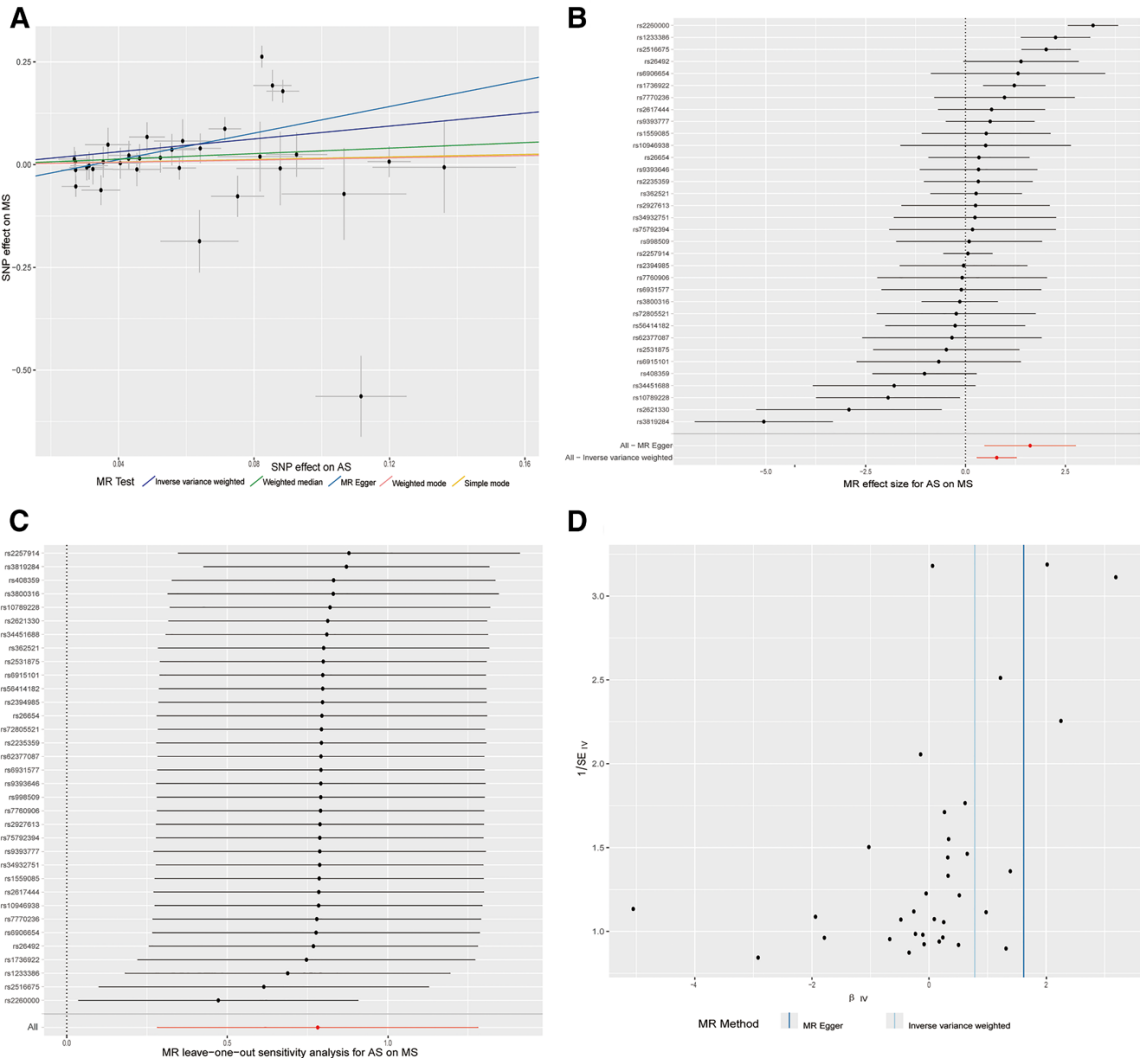
Robustness of Mendelian randomization (MR) results. This figure presents the results of the robustness tests performed to validate the MR findings. (A) The scatter plot shows the effects of SNPs on AS across different MR methods, highlighting the variation in estimates. (B) The forest plot reveals that several SNPs have confidence intervals crossing 1 (the null effect value), indicating no strong or consistent causal relationship with AS. However, certain SNPs show notable deviations, suggesting potentially significant genetic effects. (C) The leave-one-out plot demonstrates the sensitivity analysis results after excluding individual SNPs. Excluding any single SNP did not drastically alter the overall effect estimates, supporting the robustness of the results. Some variations near effect size values of 0.5 suggest that certain SNPs may have a more significant impact on the overall estimates. (D) The funnel plot compares the MR Egger and IVW methods, showing differences in effect estimates. While both methods show similar trends, MR Egger exhibits more variability, indicating sensitivity to potential genetic effects or outliers. These robustness tests indicate that the MR results are generally reliable, with no single SNP disproportionately influencing the outcome. IVW = inverse variance weighted, SNP = single nucleotide polymorphism.

We further validated the robustness of the results, as shown in Figure 4. The scatter plot presents the MR test results, showing the effects of SNPs on AS across different MR methods. The forest plot shows that several SNPs have CIs that cross 1 (the null effect value), indicating no strong or consistent causal relationship with AS. However, certain SNPs show notable deviations, both in positive and negative directions, which suggest potentially significant genetic effects. The leave-one-out plot displays the sensitivity analysis results after excluding individual SNPs. The plot indicates that excluding any individual SNP did not drastically alter the overall effect estimates, supporting the robustness of our findings. Nonetheless, there are some variations in the estimates, particularly around values close to 0.5, suggesting that certain SNPs may influence the effect size more significantly than others. The funnel plot compares the MR Egger and IVW methods, highlighting the differences in effect estimates. Although both methods show similar trends, the MR Egger method exhibits more variability in the estimates, indicating that it may be more sensitive to potential genetic effects or outliers. These robustness tests demonstrate that the MR results remain generally robust, with no single SNP disproportionately influencing the outcome.

In this study, we assessed heterogeneity using Cochran *Q* statistic across different methods (Table S1, Supplemental Digital Content, https://links.lww.com/MD/Q125). The results showed significant heterogeneity for the MR Egger and IVW methods (*P* < .05). This indicates that there were substantial differences in effect sizes across the IVs in these methods, which could impact the accuracy of causal inference. Additionally, no significant pleiotropy was detected in the Egger regression analysis (*P* > .05), suggesting that the pleiotropic effects of the selected IVs had a minimal impact on the results. Despite the presence of significant heterogeneity in some methods, pleiotropy had little interference with causal inference.

**Figure 5.**
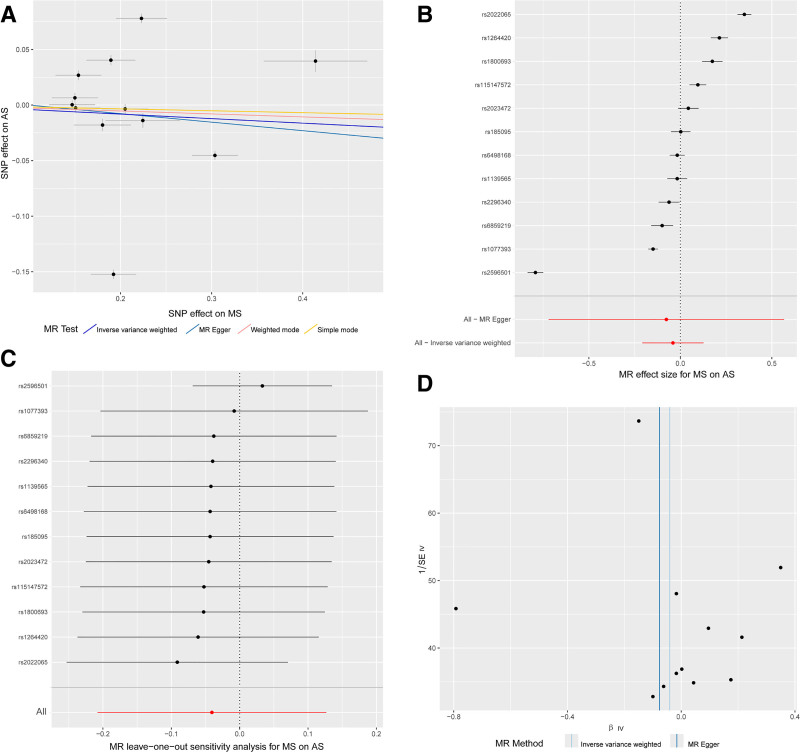
Robustness of inverse Mendelian randomization (MR) results. This figure validates the robustness of the inverse MR results. (A) The scatter plot displays the effects of SNPs on MS, with several MR methods indicating a stable association between genetic variation and MS. (B) The forest plot shows that the confidence intervals of most SNP effects cross 1 (the null effect value), suggesting minimal impact of most SNPs on MS. However, some SNPs show significant effects, indicating substantial genetic influence on MS. (C) The leave-one-out plot reveals that removing any individual SNP did not significantly alter the overall results, supporting the robustness of the findings. However, certain SNP effects, particularly around the −0.25 position, exhibited more sensitivity. (D) The funnel plot compares MR Egger and inverse variance weighted methods. While both methods display similar trends, MR Egger estimates show more fluctuation, indicating greater sensitivity to potential outliers. These robustness checks demonstrate that the inverse MR results are reliable, with no single SNP disproportionately influencing the outcome, although some SNPs exhibit greater sensitivity. MS = multiple sclerosis, SNP = single nucleotide polymorphism.

We further validated the robustness of the inverse MR results, as shown in Figure 5. Panel A displays the effects of SNPs on MS, with several methods indicating a stable association between genetic variation and MS. In the forest plot, the CIs of most SNP effects cross 1 (the null effect value), suggesting that the majority of SNPs have a minimal impact on MS. However, some SNPs show significant effects, indicating that certain genetic variations have a substantial influence on MS. The leave-one-out plot shows that removing any individual SNP did not significantly alter the overall results, suggesting the findings are robust. However, some SNP effects were more sensitive, particularly around the −0.25 position. The funnel plot compares the MR Egger and IVW methods. Although both methods display similar trends, the MR Egger estimates fluctuate more, indicating that it is more sensitive to outliers. These robustness checks demonstrate that the inverse MR results are reliable, with no single SNP disproportionately affecting the outcome, although some SNPs show sensitivity to the results.

## 4. Discussion

In this study, we used MR and inverse MR to explore the genetic relationship between AS and MS. MR analyses indicated that AS may be a genetic risk factor for MS. The IVW method yielded an OR of 2.18 (95% CI: 1.31–3.63, *P* = .00), and this result was supported by the MR Egger method. However, the weighted median, simple mode, and weighted mode methods did not detect a significant relationship, possibly due to differences in sensitivity. In inverse MR analysis, no causal relationship between MS and AS was found. All methods had *P*-values >.05, suggesting that genetic variation in MS does not influence AS. These results suggest that, at the genetic level, AS may act as a risk factor for MS, but not vice versa. To validate the robustness of our results, we performed several sensitivity analyses. These tests confirm that both the MR and inverse MR results are reliable, with no single SNP disproportionately influencing the outcomes. However, some SNPs showed sensitivity, underscoring the need for further investigation into specific genetic variations that may play a role in the development of AS and MS.

The causal association between AS and MS may be mediated by shared immunopathological mechanisms, one of which involves the IL-17 signaling pathway. Clinical observations have shown that Secukinumab, an IL-17A inhibitor, can alleviate symptoms in patients with comorbid AS and MS, suggesting that Th17-mediated inflammation may play a crucial role in the pathogenesis of both diseases.^[23]^ Moreover, increasing evidence has shown that the microbiome has a crucial role in determining the development of acquired immune responses and thereby might predispose to immune-mediated inflammatory diseases, including AS and MS. These findings imply that immune regulation associated with AS might have detrimental effects on CNS integrity, thereby increasing susceptibility to MS. In addition to pharmacological evidence, genomic studies have revealed partially overlapping susceptibility loci between AS and MS, particularly in genes involved in Th17 cell differentiation, such as IL23R, STAT3, and TYK2.^[29]^ Furthermore, the wide-spread imbalance between Th17 and regulatory T cells observed in both diseases supports a common autoimmune mechanism.^[30]^ It has also been proposed that the chronic systemic inflammation associated with AS may enhance blood–brain barrier permeability or activate microglial cells, thereby triggering immune responses against the central nervous system in genetically predisposed individuals.^[31]^

Our study’s key strength lies in the application of MR analysis, which addresses confounding factors that traditional observational studies often fail to control. By using genetic variations as IVs, MR ensures that these variations, being fixed and randomly assigned, are less influenced by external factors such as lifestyle, environment, and socioeconomic status, thereby supporting a clearer, forward causal inference and preventing inverse causation.^[32]^ MR also offers a more precise evaluation of how exposure affects health outcomes, excluding the influence of confounding variables.^[20,29]^ However, several challenges remain, particularly regarding the confounding factors involved in the selection of genetic IVs.^[20]^ It is important to note that some SNPs may influence other phenotypes potentially related to MS, even though these phenotypes have not been extensively studied, thus introducing unmeasured confounding factors. Age differences may also lead to variations in exposure levels, potentially distorting the causal relationship between exposure and outcome. In addition to age, the higher incidence of MS in females warrants consideration of gender as a potential confounder. Furthermore, this study predominantly includes individuals of European descent, which may limit the generalizability of the findings to other racial groups.^[33]^ Factors such as socioeconomic status, lifestyle, and other health conditions may also serve as potential confounders, affecting the interpretation of the results. At the statistical level, traditional MR methods assume a linear relationship between exposure and outcome, which may overlook potential nonlinear associations.^[34]^

## Acknowledgments

We thank the German Competence Network Multiple Sclerosis project and the Wellcome Trust Case Control Consortium 2 project for providing the summary data used in this study. We are very grateful to the School of Mathematics and Statistics for reviewing the statistical data of the manuscript.

## Author contributions

**Data curation:** Rui Liu.

**Formal analysis:** Gang Peng, Yongbing Xiao.

**Funding acquisition:** Rui Liu, Yongbing Xiao.

**Investigation:** Yongbing Xiao.

**Methodology:** Rui Liu.

**Project administration:** Yongbing Xiao.

**Supervision:** Yongbing Xiao.

**Validation:** Yongbing Xiao.

**Visualization:** Rui Liu.

**Writing – original draft:** Rui Liu, Gang Peng.

**Writing – review & editing:** Yongbing Xiao.

## Supplementary Material


