# Supplementary material for: Causal relationship between ankylosing spondylitis and multiple sclerosis: Evidence from 2-sample Mendelian randomization
Source: Medicine (Baltimore). 2025 Sep 19;104(38):e44788. doi: 10.1097/MD.0000000000044788 (PMC12459516; doi:10.1097/MD.0000000000044788)
Supplement: Supplementary file 1 [file medi-104-e44788-s001.docx]

| Exposure | Outcome | Method | nSNP | Beta | SE | *p* | Q | Q_df | Q_*p* | Egger_intercept | *p*-ple |
| --- | --- | --- | --- | --- | --- | --- | --- | --- | --- | --- | --- |
| AS | MS | MR Egger | 34 | 1.62 | 0.58 | 0.01 | 169.7 | 32 | 0 | -0.05 | 0.12 |
|  |  | Weighted median | 34 | 0.33 | 0.21 | 0.1 |  |  |  |  |  |
|  |  | Inverse variance weighted | 34 | 0.78 | 0.26 | 0 | 182.96 | 33 | 0 |  |  |
|  |  | Simple mode | 34 | 0.16 | 0.33 | 0.64 |  |  |  |  |  |
|  |  | Weighted mode | 34 | 0.14 | 0.34 | 0.69 |  |  |  |  |  |

Table S1: MR Analysis Results for AS and MS Using Different Methods, Including Q-test and Pleiotropy Assessment.

AS, ankylosing spondylitis; MS, multiple sclerosis; SNP, single nucleotide polymorphisms; SE, standard error; Q, Q test; Q_df, the degrees of freedom for the Q test; Q_*p*, *p*-value for the Q test; *p*-ple, *p*-value for pleiotropy.
